# Cryptic-site-specific antibodies to the SARS-CoV-2 receptor binding domain can retain functional binding affinity to spike variants

**DOI:** 10.1128/jvi.01070-23

**Published:** 2023-11-29

**Authors:** Kan Li, Richard H. C. Huntwork, Gillian Q. Horn, Milite Abraha, Kathryn M. Hastie, Haoyang Li, Vamseedhar Rayaprolu, Eduardo Olmedillas, Elizabeth Feeney, Kenneth Cronin, Sharon L. Schendel, Mark Heise, Daniel Bedinger, Melissa D. Mattocks, Ralph S. Baric, S. Munir Alam, Erica Ollmann Saphire, Georgia D. Tomaras, S. Moses Dennison

**Affiliations:** 1 Center for Human Systems Immunology, Duke University, Durham, North Carolina, USA; 2 Department of Surgery, Duke University, Durham, North Carolina, USA; 3 Center for Infectious Disease and Vaccine Research, La Jolla Institute for Immunology, La Jolla, California, USA; 4 Duke Human Vaccine Institute, Duke University, Durham, North Carolina, USA; 5 Department of Genetics, University of North Carolina, Chapel Hill, North Carolina, USA; 6 Carterra Inc., Salt Lake City, Utah, USA; 7 Department of Microbiology and Immunology, University of North Carolina, Chapel Hill, North Carolina, USA; 8 Department of Epidemiology, University of North Carolina, Chapel Hill, North Carolina, USA; 9 Department of Pathology, Duke University, Durham, North Carolina, USA; 10 Department of Integrative Immunobiology, Duke University, Durham, North Carolina, USA; 11 Department of Molecular Genetics and Microbiology, Duke University, Durham, North Carolina, USA; Loyola University Chicago - Health Sciences Campus, Maywood, Illinois, USA

**Keywords:** SARS-CoV-2, COVID-19, RBD, binding kinetics, epitope binning, monoclonal antibodies, neutralizing antibodies, surface plasmon resonance, biolayer interferometry, ACE-2 blocking

## Abstract

The COVID-19 pandemic, caused by the SARS-CoV-2 virus, has accumulated more than 700 million infection cases and 6.9 million deaths. New variants have affected antibody interaction with the surface spike protein. We defined the domain specificities and measured hexa-proline stabilized spike protein (HexaPro) binding kinetics of a large panel of antibodies sourced by the Coronavirus Immunotherapeutics Consortium. Epitope binning analysis of antibodies competing for HexaPro binding separated the fine specificities of the majority of antibodies to four regions: top, outer, mesa/valley, or cryptic site of receptor binding domain (RBD). Most of the top-RBD-specific antibodies showed >3-fold loss of binding and authentic-virus neutralization activity for the B.1.351 variant. Remarkably, among RBD mesa/valley-specific or cryptic-site-specific antibodies, 55% showed >3-fold stronger affinities, and at least 60% maintained neutralization activity for the B.1.351 variant. These data also highlighted the diversity of SARS-CoV-2-specific antibodies that retain high spike affinities and antiviral functions across variants.

**IMPORTANCE:**

Multiple SARS-CoV-2 variants of concern have emerged and caused a significant number of infections and deaths worldwide. These variants of concern contain mutations that might significantly affect antigen-targeting by antibodies. It is therefore important to further understand how antibody binding and neutralization are affected by the mutations in SARS-CoV-2 variants. We highlighted how antibody epitope specificity can influence antibody binding to SARS-CoV-2 spike protein variants and neutralization of SARS-CoV-2 variants. We showed that weakened spike binding and neutralization of Beta (B.1.351) and Omicron (BA.1) variants compared to wildtype are not universal among the panel of antibodies and identified antibodies of a specific binding footprint exhibiting consistent enhancement of spike binding and retained neutralization to Beta variant. These data and analysis can inform how antigen-targeting by antibodies might evolve during a pandemic and prepare for potential future sarbecovirus outbreaks.

## INTRODUCTION

The first cases of COVID-19 were reported in December 2019. COVID-19 was declared a global pandemic by the World Health Organization (WHO) in March 2020. Vaccines were developed and administered to populations worldwide, including three vaccines that were approved or authorized for emergency use in the United States (1). However, as the virus continues to circulate in the human population, several variants of concern (VOCs) emerged and continue to cause new and breakthrough infections (2–6). Therefore, it is important to understand how these VOCs have influenced the effectiveness of immune response and previously employed prevention strategies.

COVID-19 infection is caused by the virus SARS-CoV-2, which is included in the sarbecovirus subgenus (7). SARS-CoV-2 contains a single-stranded RNA inside its membrane. The predominant surface protein is the spike (S) protein (8, 9). The S protein is trimeric, with each protomer consisting of an S1 and an S2 subunit. The S1 subunit can be further divided into four separate domains, including the N-terminal domain (NTD) and receptor binding domain (RBD) (10). The receptor binding motif (RBM) of RBD can bind to the angiotensin-converting enzyme-2 (ACE-2) receptor on human airway epithelial cells, triggering separation of S1 and S2 subunits, membrane fusion, and subsequent infection steps (11).

S protein can undergo drastic domain conformational changes. Most notably, RBD can be in either the open or the closed conformation (12). Only the open conformation is compatible with ACE-2 binding, with the ACE-2 binding site overlapping with the top of RBD (13). The three RBDs in a trimer can co-exist in open or closed conformation for each RBD.

Both RBD and NTD can be targeted by neutralizing monoclonal antibodies (mAbs). While neutralizing NTD-targeting antibodies were shown to primarily target a specific NTD supersite (14–16), existing literature prevalently separate RBD-targeting mAbs into Class I to Class IV (17, 18): Class I refers to mAbs that target binding sites that largely overlap with RBM and are only accessible when RBD is in the open conformation, with IGHV3-55 heavy chain antibodies typically targeting this site (19, 20); Class 2 refers to mAbs that target the top of RBD and can typically bind to RBD in either the open or closed conformation; Class 3 refers to mAbs that bind more outwards onto RBD than Class 2, with S309 being one of the extreme cases (21); Class 4 refers to mAbs that bind to the inner/cryptic site of RBD (22), which is only accessible when RBD is in open conformation, with CR3022 mAb being a representative member of this class (23).

There are other variations of binding site classification. Class 3 encompasses a large variety of epitopes arranged along the outer side of RBD. Yuan et al. separated Class 3 into three smaller groups, depending on the distance of the epitope from RBM (20, 24). Deshpande et al. defined a new group called “Class I distinct,” which is essentially a sub-group of Class 2 that binds between the ACE-2 binding site and the center-top area of RBD (25). Dejnirattisai et al. used a separate nomenclature and compared RBD to a human torso (26), essentially separating the top of RBD into smaller areas as well as further separating Class 3 binders.

Several variants of concern (VOCs) emerged, including B.1.1.7 (Alpha), B.1.351 (Beta), P.1 (Gamma), B.1.617.2 (Delta), and B.1.1.529 (Omicron), and each was at a certain stage of the pandemic rapidly transmitted among the human population. Among the key mutations, mutation N501Y is shared by B.1.1.7, B.1.351, P.1, and B.1.1.529 and was shown to be the crucial mutation to enhance the affinity to ACE-2 (27, 28). Mutations of K417 and E484 are shared by B.1.351 (K417N/E484K), P.1 (K417T/E484K), and B.1.1.529 (K417N/E484A). The mutation of K417N/T alone abolishes the stabilizing salt bridge formed by the Lysine and is unfavorable for ACE-2 binding (27, 28). The mutation on position E484 does not significantly impact ACE-2 binding affinity (29, 30), although the structure of the unbound spike containing E484K mutation suggests the mutation has a role in the local destabilization of RBD structure (31). The combination of the three mutations in B.1.351 and P.1 has a net effect of increasing RBD affinity to ACE-2 (28, 30).

Administering antibody cocktails is a great option to target multiple antigen epitopes simultaneously. Several commercially available antibody cocktails were developed to treat individuals with COVID-19 (21, 32–39). Additional cocktail formulations have also been proposed by other research groups (40–42). All major clinical mAbs under clinical trials and previously under FDA emergency use authorization (EUA) (43) (Bamlanivimab, Etesivimab, Casirivimab, Imdevimab, Cilgavimab, Tixagevimab, Amubarvimab, Romlusevimab, Bebtelovimab, Regdanvimab, Sotrovimab, and Adintrevimab) can be classified as Classes 1–3, but none are Class 4 (cryptic site) mAbs that bind similar to CR3022 (44, 45).

Class I and class II antibodies predominantly form stabilizing contacts with K417 and E484, respectively (20, 46, 47). Therefore, a significant number of clinically available mAbs showed weaker binding or neutralization to VOCs due to loss of stabilizing interactions (48–53). It is thus important to further characterize the impact of the binding affinity of mAbs due to variant mutations. This will help elucidate how antigen-targeting by antibodies might evolve during a pandemic and prepare for potential future sarbecovirus outbreaks.

The Coronavirus Immunotherapeutics Consortium (CoVIC) collected more than 400 candidate therapeutic antibody constructs (IgG and other forms) to study their biophysical and functional properties and their resistance to VOCs. The first 300 antibody constructs were collected prior to the emergence of the Delta variant while the remaining antibody constructs were collected by the fall of 2022 and at least include antibodies isolated from individuals infected with the Delta variant. The majority of the RBD-specific CoVIC constructs were separated into seven major communities based on competition profiles against isolated RBD domain molecules (54). These communities encompass the four epitope classes (17, 18), with further separation into sub-communities for each class. In particular, a specific area on the top of RBD termed the “mesa” area, similar to the “right shoulder” in the human torso analogy (26), is shared by multiple communities as part of the binding interface. Representative CoVIC constructs were then selected from each community for further structural and functional studies, including characterizing the RBD target site(s) for each community as well as assessing the impact of variant mutations on neutralization.

When RBD is presented as an integral part of the trimeric S protein, the competition profile may change due to factors such as steric hindrance and the open/closed state of RBD (12). Here, we report the competition profile for CoVIC constructs against HexaPro, a trimeric prefusion-stabilized S protein containing six stabilizing proline residues (55), using an epitope binning method by high-throughput surface plasmon resonance (SPR), and how this competition profile relates to binding affinities and ACE-2 blocking ability of CoVIC antibodies. We also report the impact of B.1.351 and BA.1 mutations on antibody affinities for the spike protein and neutralization function.

## RESULTS

### Majority of constructs in the CoVIC panel are RBD specific

To examine the domains within HexaPro targeted by the CoVIC antibody constructs and to determine the binding affinities, we carried out high-throughput SPR binding kinetics assays of RBD, NTD, and D614 HexaPro proteins interacting with immobilized CoVIC antibody constructs. A majority (76%) of the CoVIC antibody constructs exhibited specificity for RBD, while a smaller fraction (11%) showed specificity for NTD. A small subset targeted neither the RBD nor the NTD (Fig. S1 and S2). This panel includes IgG molecules as the majority of its members but also includes bispecific antibodies, non-IgG types including IgY molecules, antigen-binding fragments (Fabs), VHH fragments, tandem VHH, and VHH-Fc fusion molecules. Due to this complexity and for inclusivity, “antibody constructs” or “constructs” are used here instead of “antibodies” when describing members of the CoVIC panel.

### Epitope binning using HexaPro binding competition profiles separates non-RBD binders from the communities of RBD binding antibodies

**Fig 1:**
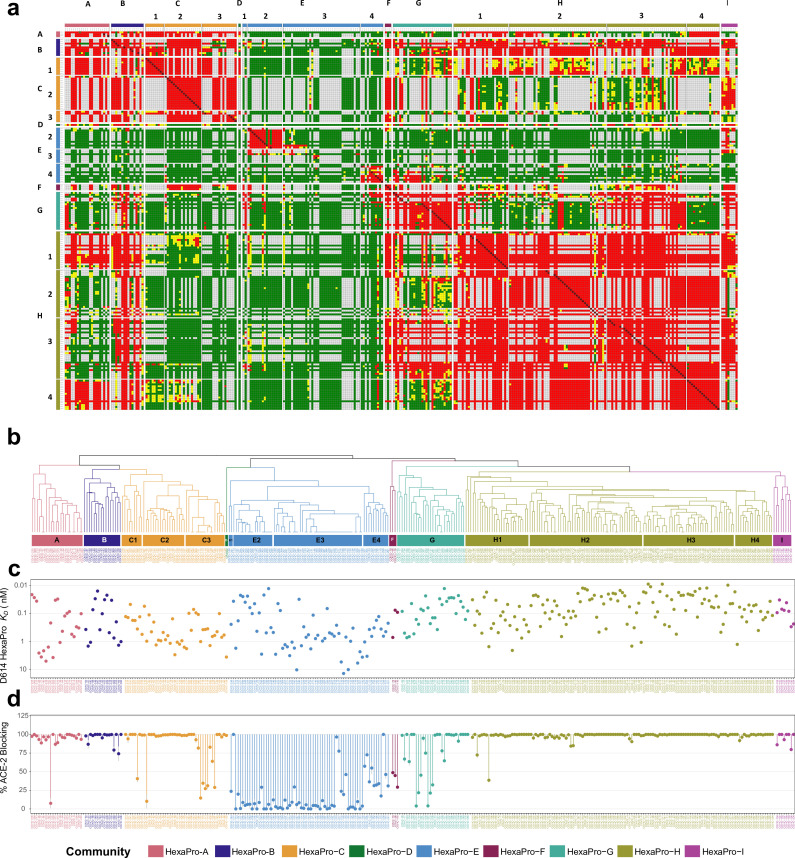
Epitope binning on HexaPro separates the CoVIC panel into nine communities, with distinct completion profile, affinity range, and ACE-2 blocking ability. (a) Competition map of HexaPro binning communities from combining the results of two binning assays. Each colored square represents the categorization of normalized response for the specific pair of CoVIC constructs: red indicates competition (<0.5); green indicates sandwiching (>0.7); yellow indicates intermediate response (≥0.5 and ≤0.7); white indicates competition pairs where the data were not measured. The CoVIC IDs on the columns and rows correspond to the CoVIC IDs of the ligands (on chip surface) and the analytes (immune complexed with HexaPro), respectively. (b) Dendrogram derived from the competition map. The naming for each community and the corresponding color are shown in the legend (e.g., HexaPro-A, HexaPro-B). Sub-clusters of HexaPro-C, -E, and -H are indicated in panels a and b. (c) Binding *K_D_* value for D614 HexaPro separated by HexaPro binning community. (d) Percent blocking of ACE-2 to RBD binding separated by HexaPro binning community, with error bars shown as gray vertical lines.

To define fine specificities of the CoVIC panel for S protein, each of CoVIC antibody constructs 1–395 was tested pairwise in a high-throughput SPR competition binding assay. The assay used the premix epitope binning format where an immune complex was pre-formed by incubating D614 HexaPro with an excess of each antibody construct and then tested for binding to antibody constructs immobilized on the SPR chip surface. Depending on whether the two antibody constructs compete for binding to the same epitope on the S protein, further binding or lack thereof by the chip surface-immobilized antibody construct to the mixture can be observed. The excess amount of antibody construct in the solution ensures saturation of the binding site. The competition heatmap (Fig. 1a) and competition dendrogram (Fig. 1b) were then generated from the responses normalized to the responses of antigen alone binding to the chip surface antibody constructs (Fig. 1; Fig. S3; Table S1). Regeneration of the chip surface was carried out before the binding of each subsequent antibody construct-HexaPro mixture. The data for some antibody constructs as (immobilized) ligands were unusable due to either inefficient regeneration or the regeneration denatured the ligand leading to a rectangle competition matrix (Fig. 1a).

Using combined competition map from two separate binning assays that included different subsets of the CoVIC panel and the deduced dendrogram (see the section Materials and Methods for details), the CoVIC panel was separated into nine communities named HexaPro-A to –I (Fig. 1b). Some of the communities were separated into sub-clusters (e.g., HexaPro-C1 to -C3, HexaPro-E1 to -E4, and HexaPro-H1 to -H4) that showed distinct competition profiles but shared common features with other sub-clusters. The non-RBD binders clustered in one community (HexaPro-E), while the RBD binders were separated into several communities. For communities containing more than two antibody constructs (HexaPro-D only contains one member, CR3022), we detail below the competition profiles, binding affinities to D614 HexaPro (Fig. 1c), and the ability to block ACE-2 from interacting with RBD (Fig. 1d).

The HexaPro-E community contains all the non-RBD-specific binders and a small group of RBD binders. The weak or absence of competition against constructs in all other communities is this community’s most notable feature. The majority of HexaPro-E antibody constructs (61/68, 89.7%) also cannot effectively block ACE-2 (0%–46%, Fig. 1d). The lack of competition and ACE-2 blocking indicates that HexaPro-E antibody constructs, including RBD-binders in HexaPro-E1 and -E4, target epitopes distal from the RBM. However, HexaPro-E constructs target a wide variety of epitopes, as indicated by the lack of competition within the community. Intra-community competition was observed only for two isolated sub-groups (NTD binders in Hexapro-E2 and RBD binders in HexaPro-E4).

HexaPro-H represents the biggest community with close to complete intra-community competition (Fig. 1a and b). Almost all the constructs in this community (128/129, 99.2%) effectively block ACE-2 (72%–100%) (Fig. 1d), indicating that the binding epitopes significantly overlap with the RBM. The majority of these constructs have sub-nanomolar binding affinities to D614 HexaPro and on average the strongest spike affinities among RBD binders (8 pM to 3 nM, average 171 pM) (Fig. 1c).

HexaPro-A and -C are closely related (Fig. 1b), with close to complete competition between the two communities (Fig. 1a). These two communities likely target significantly overlapping epitopes. Among communities of RBD binders (HexaPro A-D, F-H), HexaPro-A and -C have on average the weakest affinities (20 pM to 6 nM, average 811 pM; Fig. 1c). HexaPro-A only compete with HexaPro-H1 and -H4 but not the other HexaPro-H sub-clusters; HexaPro-C2 and -C3 shows close to no competition with HexaPro-H. With a lack of community-wide competition against HexaPro-H, and with the majority of the constructs strongly blocking ACE-2 (>80% blocking) (Fig. 1d), HexaPro-A and HexaPro-C constructs are expected to target epitopes that overlap with RBM from a different side of RBD than the footprint occupied by HexaPro-H. HexaPro-C1 shows community-wide competition asymmetry: HexaPro-H constructs as analytes (*i.e.*, complexed with HexaPro) block HexaPro binding to constructs of HexaPro-C1 immobilized on the chip (ligands). However, no blocking was observed in the reverse orientation (Fig. 1a). This might indicate that HexaPro-C1 represents footprints that are more easily buried by steric hindrance. Therefore, HexaPro-A and -C footprints are likely closer to the inner side of RBD than those of HexaPro-H constructs.

HexaPro-B shows community-wide strong competition against HexaPro-H (Fig. 1a). On average, HexaPro-B constructs show intermediate binding affinities to D614 HexaPro among RBD binders (15 pM to 2 nM, average 464 pM; Fig. 1c). All HexaPro-B constructs effectively block ACE-2 (74-100%; Fig. 1d), indicating these constructs target epitopes that overlap with RBM. In contrast to HexaPro-H, HexaPro-B showed close to complete competition against HexaPro-A and -C. Therefore, HexaPro-B resembles HexaPro-H in terms of binding affinity and ACE-2 blocking but exhibits a different competition profile. Overall, HexaPro-B shows strong to complete competition with all communities of RBD binders (HexaPro A-C, F-H) except for HexaPro-D which contains only CR3022, indicating that the epitope footprint of HexaPro-B constructs may overlap with those of all other main RBD communities. The extent of overlap could be different depending on the specific community.

HexaPro-F and -G are closely related (Fig. 1b), with close to complete competition between the two communities (Fig. 1a). HexaPro- F and -G constructs show a wide range of ACE-2 blocking ability, with most of them (26/32, 81.3%) showing intermediate to strong ACE-2 blocking (45-100%; Fig. 1d). HexaPro-F and -G both compete strongly with HexaPro-H3. HexaPro-F shows competition asymmetry: some HexaPro-F constructs as analytes (i.e., complexed with HexaPro) block HexaPro binding to constructs of HexaPro-H1 immobilized on the chip (ligands). However, no blocking was observed in the reverse orientation (Fig. 1a). This might indicate that HexaPro-F and -G footprints lie outwards than those of HexaPro-H.

HexaPro-I constructs are majority bispecific, indicated by their competition to several RBD communities as well as to NTD binders in HexaPro-E2 (Fig. 1a). Constructs in this community might also bind to RBD footprints that overlap with those for most of the other RBD communities, for HexaPro-I constructs show strong competition to all RBD communities except HexaPro-A.

**Fig 2:**
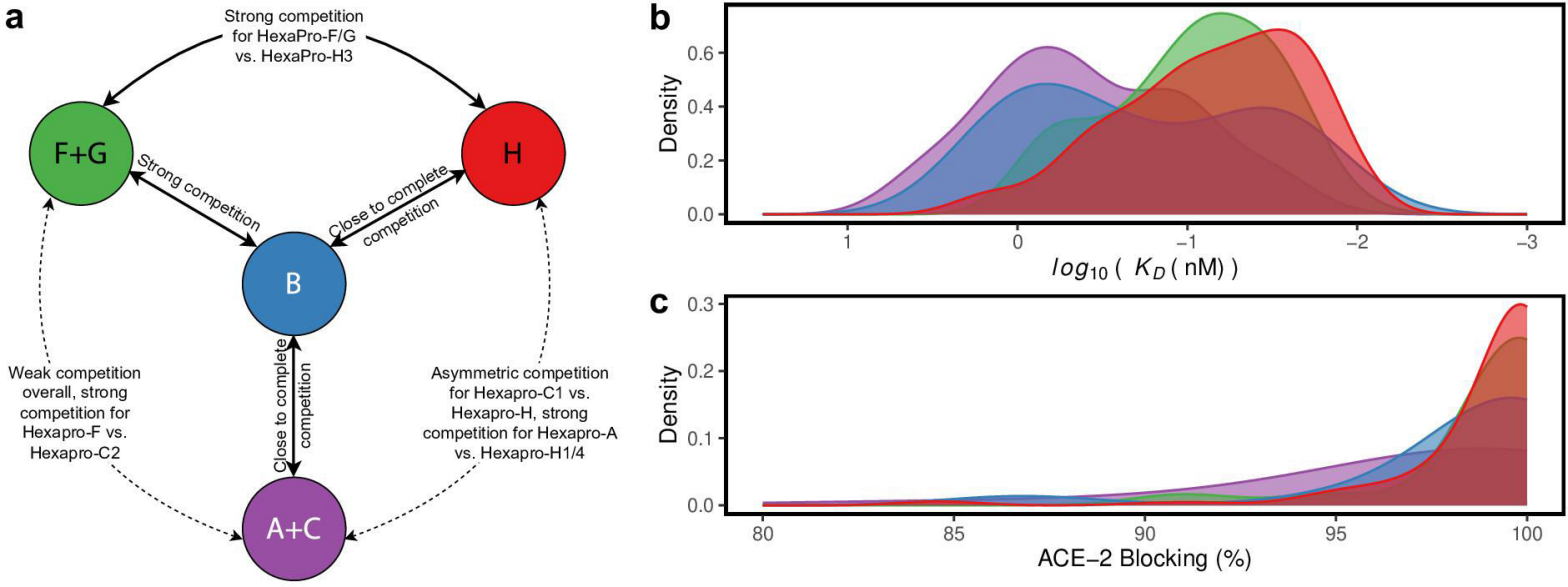
RBD-binder communities with more than two members can be separated into four categories. (a) A cartoon summary of the competition relationship among the four RBD-binding community categories is shown. Black texts indicate the competition relationship between each pair of community categories. (**b-c**) Distribution of *K_D_* values for binding to D614 HexaPro (b) and percentage ACE-2 blocking (c) of CoVIC constructs in each of the four categories. The color code corresponds to the color scheme in (a).

Overall, the RBD binder communities with more than two members (HexaPro A-C, F-H) can be separated into four categories (Fig. 2): HexaPro-F and -G likely represent a group that has the most outward binding footprint on RBD. HexaPro-H epitopes overlap significantly with RBM and the constructs bind to D614 HexaPro strongly. Most constructs in HexaPro-A and –C also overlap with RBM but have different and likely more inward binding footprints than HexaPro-H. HexaPro-B epitopes overlap with RBM and epitopes for most RBD binders, indicating the binding footprint is a type of nexus for the other footprints.

### The CoVIC constructs that target the top and cryptic site of RBD are clustered similarly when binned against HexaPro and isolated RBD protomer

**Fig 3:**
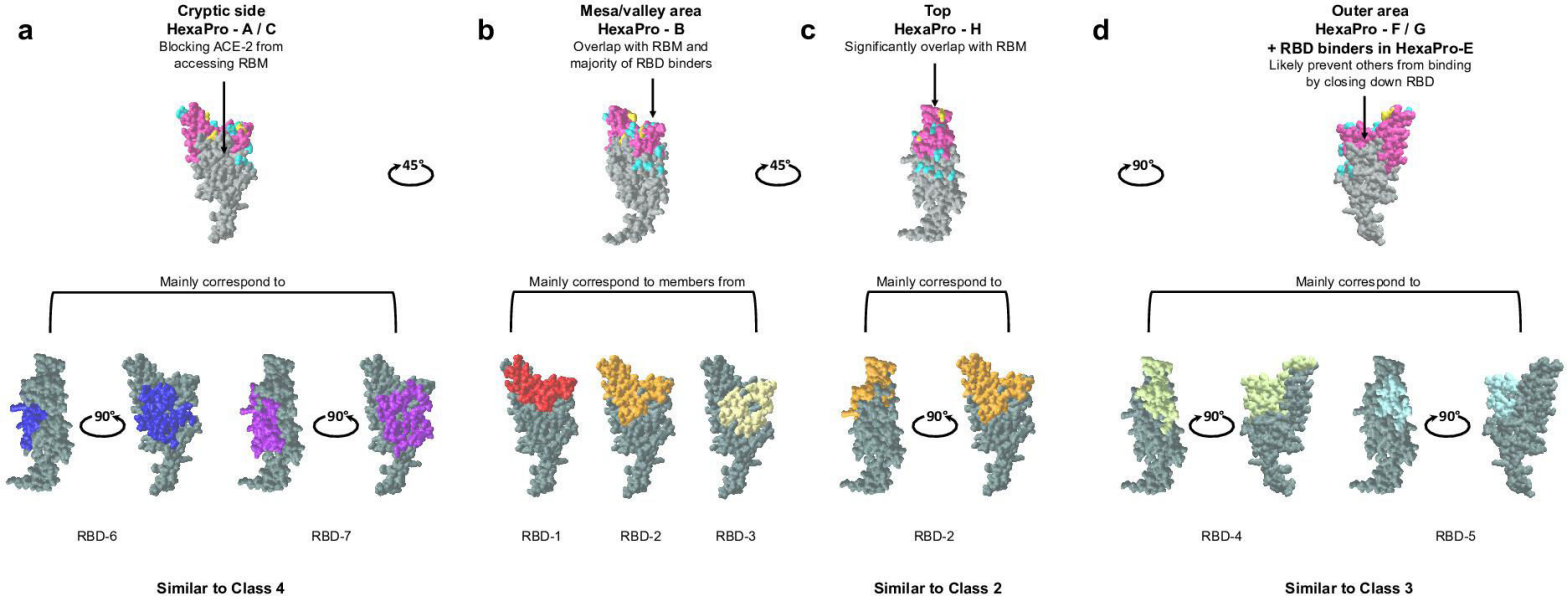
Antibody constructs that target the top, cryptic side, and outer area of RBD can be grouped similarly among HexaPro binning communities, RBD binning communities, and the conventional classification system. (a**-d**) Illustration of the four main epitope footprints on RBD surface based on HexaPro epitope binning results. In the top row, RBM is colored in dark pink, RBD mutated residues in B.1.351 variant are colored in gold, and RBD mutated residues in BA.1 but not in B.1.351 are colored in cyan. In the bottom row, footprint illustrations of the main corresponding RBD communities and the corresponding conventional classifications are indicated for each footprint. RBD surface and footprint illustrations were generated from CoVIC-DB (https://CoVICdb.lji.org/) using PDB 7DDD (57).

The majority of the CoVIC antibody constructs are RBD specific (Fig. S2). To understand the similarities and differences in the clustering pattern of CoVIC antibody constructs when binned against the D614 HexaPro versus against the isolated RBD protomer, we compared the community separation of HexaPro binning against that of RBD binning previously done for the CoVIC panel (54, 56) (Fig. 3; Fig. S4).

The majority of constructs (112/130, 86%) in the HexaPro-H were grouped in the same RBD bin, RBD-2 (Fig. S4). Structural data for representative RBD-2 constructs (54) indicate that these constructs bind to the top of RBD (Fig. 3c), similar to Class 2 (17, 18, 20, 25), accounting for strong ACE-2 blocking by HexaPro-H. The majority of binders in HexaPro-H1, -H2, and -H4 were clustered in RBD-2a and −2b, whereas all binders in HexaPro-H3 were clustered in RBD-2c and -2d except one (Fig. S4). All four RBD-2 subclusters were shown to occupy the peak of RBD but overlap differently with RBM (54), with RBD-2c and -2d footprint closer to the outer side of RBD. Here, the peak of RBD is very similar to the “left shoulder” defined by Dejnirattisai et al. (26).

All constructs in HexaPro-C were grouped in the same RBD bin (RBD-7) while the majority of constructs in HexaPro-A (19/22, 86.4%) were grouped in RBD-6/7 (15/22, 68.2%) and RBD-3 (4/22, 18.2%) (Fig. S4). Structural data on representative constructs for RBD-6 and −7(54) indicate that these constructs bind to the cryptic site of RBD (Fig. 3a), similar to Class 4 (17, 18, 22, 23). The HexaPro binning competition shows that HexaPro-H and HexaPro-A/C use different binding footprints to block ACE-2, which is supported by the structural data (54). HexaPro-A strongly competes with HexaPro-H1/H4 (Fig. 1a), indicating that these two subsets bind to footprints that are closest in terms of RBD-top versus RBD cryptic site.

The majority of constructs (30/32, 93.8%) in HexaPro-F and -G were grouped in RBD-4 and -5 (Fig. S4), which are indicated by representative structural data (54) to target the outer face of RBD (Fig. 3d), similar to Class 3. Therefore, it is reasonable that HexaPro-F/G constructs can prevent RBD-top binders from accessing their binding interface. For example, some outer face binders can bind to both open and closed RBD but might prevent other constructs from binding by locking RBD in the closed state, similar to previously reported mAbs (58, 59). HexaPro-F/G strongly competes with HexaPro-H3 (Fig. 1A), with HexaPro-H3 targeting top binding footprints that are closer to the outer side than other HexaPro-H subclusters, also validating the binding preference of HexaPro-F/G. Interestingly, the sub-group of RBD-binders in HexaPro-E4 showing self-blocking was grouped in a specific RBD-5 sub-cluster (RBD-5b) (Fig. S4), suggesting that these constructs may target outer face footprints far removed from RBM and cannot lock RBD in the closed state, resulting in no competition against other RBD binders.

Members in HexaPro-B belonged to a number of RBD communities in Hastie et al. and Callaway et al. (54, 56) (Fig. 3; Fig. S4). The majority (13/16, 81.3%) were grouped in RBD-1, -2, and -3. These constructs target different RBD footprints but share the mesa area on the opposite side of the RBD peak, similar to the “right shoulder” defined by Dejnirattisai et al. (26), as well as the valley between the mesa and peak, as part of the binding footprints. Therefore, Hexapro-B constructs do not directly correspond to any of the four classes. It is possible that the surrounding domains in HexaPro trimer can restrict how RBD-specific antibodies access binding sites, especially the cryptic site and outer face. This restricted access in addition to the RBD mesa’s close proximity to the RBD peak may allow constructs sharing mesa/valley area for binding footprints to effectively compete against the majority of RBD binders that bind to the top, inner, and outer sides.

HexaPro-C1, -C2, and -C3 showed further differences when simultaneously considering ACE-2 blocking and competition against HexaPro-H (Fig. 1a and d). HexaPro-C1 constructs effectively block ACE-2 (94%–100% for all except one) and show asymmetric competition with top side binders (HexaPro-H). HexaPro-C2 and -C3 show close to no competition against top side binders in HexaPro-H. However, HexaPro-C2 constructs effectively block ACE-2 (98%–100% for all except one) while HexaPro-C3 constructs show a wide range of ACE-2 blocking abilities (15%–100%). This suggests that HexPro-C1 binders target footprints that are easily sterically blocked by the binding of RBD-top, while HexPro-C3 represents a group targeting footprints further away from the ACE-2 binding site. These differences indicate that the footprints targeted by this cryptic-site binding community can be separated further into at least three smaller regions.

Overall, CoVIC antibodies and antibody constructs were separated into communities that target the top (H), outer area (F, G, and RBD-binders in E), cryptic site (A and C), and mesa/valley area of RBD (B) (Fig. 3) as well as one community that encompasses almost all non-RBD binders (E) and one community that encompasses bi-specific binders (I). Separation of the RBD binders into these four groups can adequately capture the main epitope specificity separation. HexaPro-B encompasses a number of RBD binders sharing the RBD mesa/valley for binding footprints, with these constructs binned in different communities when using isolated RBD molecules.

### B.1.351 mutations weaken affinities of top-targeting CoVIC antibodies and constructs while strengthening those targeting the cryptic site

**Fig 4:**
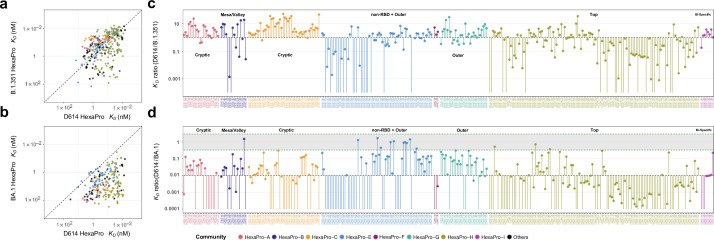
RBD-inner face binders (HexaPro-A and -C) consistently show enhanced affinity for B.1.351 HexaPro relative to D614 HexaPro and have less significant loss of affinity for BA.1 HexaPro relative to D614 HexaPro when compared to RBD-top specific binders (HexaPro-H). (a, b) *K_D_* value correlation between D614 HexaPro and B.1.351 HexaPro (a) or between D614 HexaPro and BA.1 HexaPro (b). The diagonal line is a theoretical line where the correlating affinities are identical. The areas above and below the diagonal line contain CoVIC IDs that have enhanced and weakened affinity, respectively, compared to D614 HexaPro. If binding by a specific CoVIC ID to B.1.351 or BA.1 is abolished, no data point for that construct is shown. The points are colored according to HexaPro community designations. CoVIC IDs without a community designation are in black. (c, d) Affinity ratios (D614 HexaPro *K_D_* / B.1.351 HexaPro *K_D_* (c) or D614 HexaPro *K_D_* / BA.1 HexaPro *K_D_* (d) organized by HexaPro epitope community. A *K_D_* ratio >1 indicates an enhanced affinity for variant HexaPro relative to D614 HexaPro; a *K_D_* ratio <1 indicates a weaker affinity for variant HexaPro relative to D614 HexaPro. In (c), each vertical line connects a ratio of 1 to the *K_D_* ratio for a given CoVIC ID. In (d), each vertical line connects a ratio of 0.01 to the *K_D_* ratio for a given CoVIC ID. A gray band in (d) with green borders indicates a range of ratio from 3 to 1/3. Lines without a capping point correspond to CoVIC constructs that had no B.1.351 or BA.1 binding.

To assess how the binding affinities of CoVIC antibodies and constructs are affected by the D614G mutation, mutations on B.1.351 variant and mutations on B.1.1.529 (BA.1) variant, with the latter two including mutation D614G, we measured the affinities of CoVIC constructs (CoVIC 1–405) for D614G HexaPro, B.1.351 HexaPro, and BA.1 HexaPro (HexaPro that contains B.1.351 or BA.1 mutations). Overall, with residue 614 very distant from RBD and NTD, D614G mutation alone expectedly does not significantly alter the binding affinities (Fig. S5), with R^2^ = 0.846 of linear regression and Rho = 0.920 (*P* < 1 × 10^−5^) of Spearman correlation. By contrast, the additional mutations on B.1.351 HexaPro did significantly change the binding affinities (Fig. 4a), with R^2^ = 0.151 of linear regression and Rho = 0.439 (*P* < 1 × 10^−5^) of Spearman correlation. Compared to D614 HexaPro, 48% of the panel showed weakened to abolished binding to B.1.351 HexaPro. BA.1 mutation further weakened the binding affinities, with only 29 constructs retaining similar binding compared to D614 and 38% of the panel abolishing binding (Fig. 4b).

When separated by Hexapro binning communities, three groups showed drastic changes in the binding to B.1.351 compared to HexaPro, as shown by the ratio of *K_D_* values (Fig. 4c).

As a group, top-RBD targeting antibody constructs, while associated with the strongest affinity range to D614 HexaPro (Fig. 1c), showed generally weakened to abolished binding to B.1.351. In HexaPro-H, the majority of constructs (105/130, 80.7%) show *K_D_* ratios below 2, meaning no significant change or a weakened to abolished B.1.351 binding. Among the 130 constructs, 67 showed a ratio below 1, and 19 showed no detectable B.1.351 binding.

Most NTD binders, while epitope specificities not yet determined, also have their binding to B.1.351 weakened or abolished. For NTD-specific constructs in HexaPro-E, 18 out of 35 showed abolished B.1.351 binding, and another eight showed more than 10-fold weaker B.1.351 binding.

By contrast, antibody constructs targeting the RBD cryptic site showed the weakest affinity range for D614 HexaPro (Fig. 1c) but showed consistently enhanced binding to B.1.351. 61 out of 66 constructs in HexaPro-A and -C show *K_D_* ratios above 1, with more than half (38/66, 57.6%) showing *K_D_* ratios above 3. The remaining 5 out of 66 show *K_D_* ratios above 0.33. When comparing BA.1 to D614 HexaPro (Fig. 4d), cryptic-site-targeting antibodies continue to show less extent of affinity weakening compared to top targeting antibodies. 50% of HexaPro-A and -C constructs showed less than 100-fold affinity weakening, compared to 23% of constructs in HexaPro-H.

Other groups exhibited minor changes in B.1.351 affinity (Fig. 4b). In HexaPro-F and -G, 28 out of 32 constructs showed 0.31- to 6.56-fold changes in B.1.351 affinities, indicating their lack of overlap with RBM despite competition with top and mesa targeting constructs. All 14 RBD-specific constructs in HexaPro-E should be far removed from RBM based on binning and showed 0.56- to 3.9-fold changes in B.1.351 affinities. Therefore, the binding of outer-RBD targeting constructs is not greatly affected by B.1.351 mutations. In addition, most HexaPro-E constructs (1619, 84%) targeting outside of RBD and NTD (Fig. S4) showed no significant change to slightly stronger affinities (0.34- to 4.5- fold). Except for one, bispecific antibodies in HexaPro-I also showed no significant change (1.0- to 3.8-fold). In all these groups, 57 out of 73 constructs showed less than 120-fold weakened BA.1 affinity, with 75% of the 57 constructs less than 10-fold weakened (Fig. 4d).

### CoVIC constructs showing enhanced affinity to B.1.351 HexaPro are more likely to retain authentic virus neutralization activity against B.1.351 VOC

**Fig 5:**
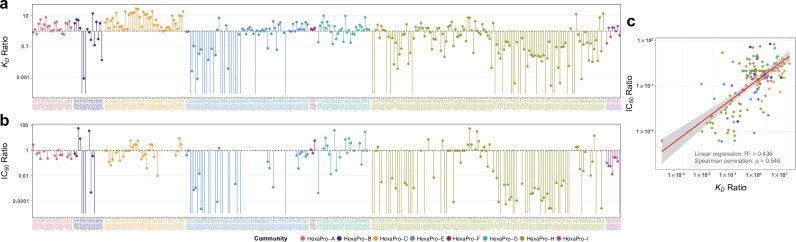
CoVIC constructs that show enhanced affinities for B.1.351 HexaPro are more likely to retain neutralization activity against the B.1.351 variant. (a) Ratios of *K_D_* values (D614G HexaPro *K_D_* / B.1.351 HexaPro *K_D_*) are shown. (b) Ratios of IC_50_ values (D614G HexaPro IC_50_ / B.1.351 HexaPro IC_50_) for authentic neutralization assays are shown. IC_50_ ratios were calculated based on values deposited in the CoVIC database contributed by the University of North Carolina (CoVIC-DB (60); see https://CoVICdb.lji.org/ for detailed values and descriptions of the method). (c) The correlation is shown between the ratio of *K_D_* values and the ratio of IC_50_ values for the constructs in panels a and b. In all panels, each CoVIC construct is colored based on its HexaPro community designation.

Next, we assessed the relationship between the changes in affinity for the B.1.351 variant in comparison to the D614G HexaPro and the corresponding changes in neutralization efficiency for the CoVIC panel. Figure 5 shows the ratios of *K_D_* values (D614G HexaPro *K_D_* / B.1.351 HexaPro *K_D_*) and ratios of IC_50_ values (D614G HexaProG IC_50_/B.1.351 HexaPro IC_50_) from authentic virus neutralization (60). There is a relatively strong correlation between the two ratios, with R^2^ = 0.436 of linear regression and Rho = 0.546 (*P* < 1 × 10^−5^) of Spearman correlation (Fig. 5c).

This correlation indicates that changes in affinity can reflect changes in neutralization to a large degree. In particular, at least 74% of cryptic-site-specific constructs maintained neutralization activity for B.1.351 (less than 10-fold weakened or enhanced) (Fig. 5b). In addition, the RBD-binding constructs showing enhanced affinity toward B.1.351 HexaPro and retained neutralization ability belong to different HexaPro binning communities. Therefore, select RBD-binding constructs in all four categories have the potential to resist B.1.351 mutations.

### Fab fragments of CoVIC constructs can better distinguish binding affinities for D614 and VOC HexaPro

**Fig 6:**
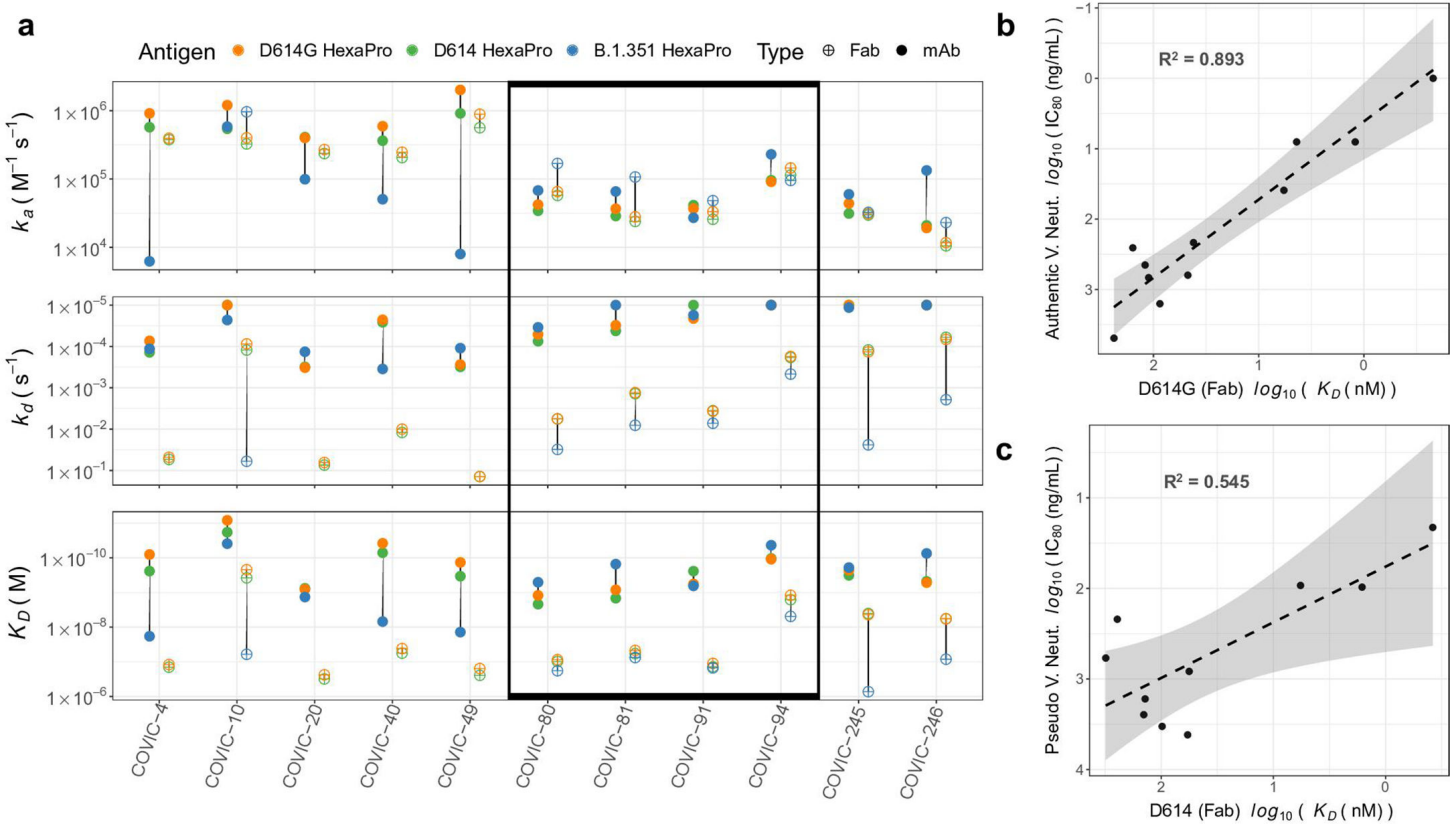
Fab fragments of CoVIC constructs targeting various binding sites can retain strong affinity to B.1.351. (a) The association rate constant (*k_a_*), dissociation rate constant (*k_d_*) and affinity values (*K_D_*) for each CoVIC construct as a mAb or Fab binding to D614, B.1.351, and D614G HexaPro. Filled and open circles correspond to values for intact IgG and Fab fragments, respectively. No value is displayed for Fab fragments that showed no detectable binding to B.1.351. Constructs showing comparable binding to all three HexaPro proteins as Fabs are indicated within the rectangle. (b) The correlation between Fab binding affinity to D614G HexaPro and the IC_80_ value for neutralization of authentic D614G virus is shown. (c) The correlation between Fab binding affinity to D614 HexaPro and the IC_80_ value for pseudovirus neutralization bearing D614 is shown. (b, c) Neutralization data were obtained from CoVIC-DB (https://CoVICdb.lji.org/). R^2^ values are from linear regression.

When monoclonal antibodies (mAbs) bind to the trimeric spike protein, their bivalent nature can contribute to the avidity effect of binding. To better characterize the correlation between binding affinities and neutralization, we produced antigen-binding fragments (Fab) of several CoVIC IgG antibodies and measured the Fab binding affinities for D614 HexaPro, D614G HexaPro, and B.1.351 HexaPro. Comparing the association and dissociation rate constants and affinities for the mAb and Fab forms of select CoVIC antibodies revealed that all Fab fragments tested bound to D614 HexaPro and D614G HexaPro, whereas only some Fab fragments bound to B.1.351 HexaPro (Fig. 6a; Fig. S6; Table S2). The constructs that lacked binding to B.1.351 HexaPro as Fabs did show binding in mAb form, but with a markedly slower association rate (>3-fold) than that of D614 HexaPro and D614G HexaPro. Generally, for each CoVIC construct, the dissociation rate increase for Fab relative to mAb for D614 HexaPro and D614G HexaPro was similar (6- to 655-fold), while B.1.351 HexaPro exhibited a much bigger dissociation rate increase (47- to 2614-fold) or a complete lack of observable binding. As a result, each CoVIC Fab generally shows a similar affinity for D614 HexaPro and D614G HexaPro (11- to 1,490-fold weaker compared to mAb), while showing much weaker binding (113- to 3,795-fold weaker compared to mAb) or no binding to B.1.351 HexaPro.

Among the Fabs tested, four constructs (CoVIC-80/81/91/94) showed comparable binding to all three HexaPro proteins (within 12.5-fold among all three Fab affinities). Based on community separations from HexaPro and RBD binning, CoVIC-81 targets the mesa/valley area, while CoVIC-94 and CoVIC-91 bind the RBD top and outer face, respectively. While CoVIC-91 showed weak neutralization to the B.1.351 variant, CoVIC-80, -81, and -94 retained both strong binding affinities and neutralization activity for B.1.351 VOC.

We next assessed whether the binding affinities of CoVIC construct Fab fragments for D614/D614G HexaPro directly correlate with neutralization activity indicated by IC_80_ values, which presented a more stringent measurement for infection inhibition level than IC_50_. Figure 6b shows the correlation between affinities for Fab binding to D614G HexaPro and the IC_80_ values from authentic virus neutralization data. Figure 6c shows a similar correlation between D614 HexaPro affinity and IC_80_ values from pseudo-virus neutralization. Linear regression showed that Fab binding affinities had an especially strong correlation with authentic virus neutralization, suggesting that Fab affinity can be a predictive measure of virus neutralization.

## DISCUSSION

Several groups have attempted to separate a panel of mAbs, Fabs, or nanobodies into groups or down-select mAbs based on competition profiles: some panels were competed against a small number of known-epitope mAbs (34, 61), some used RBD to probe competition profile (26, 62, 63), while the majority used spike trimer to probe competition profile (15, 32, 64–68). Although the epitope classification of classes 1–4 is the most prevalently adopted nomenclature and provides a broad-stroke separation of antibody binding specificities, the competition assays done here and by others show the complexity of the epitope targets. When interpreting binning results, it is important to understand how the antigen molecule used impacts the competition profile and to combine other types of data to determine the most appropriate categorization. Here, we have attempted to provide a more comprehensive landscape of binding epitopes using a large antibody panel and linking the binning communities to previously reported structural data, binding affinity changes, and neutralization ability changes. The biophysical and functional data enabled us to better define the binding footprint and characteristics each binning community represents. We also compared the competition profile probed using a spike trimer to previously reported competition using RBD (54) and showed that varying antigen probes may lead to differences in the competition profiles. In the context of the trimeric spike, epitope accessibility on RBD will vary due to steric hindrance from surrounding domains or the opening and closing of RBD, influencing competition outcome. Expectedly, the CoVIC antibody constructs separated into nine communities when binned against HexaPro trimer compared to seven main communities identified by RBD binning, although the non-RBD binders segregated away from the exclusively RBD-binding communities.

While we separated the binding footprints into multiple regions based on distinct competition patterns, the different RBD binding footprints are very close spatially as indicated by the competition among RBD-binding communities. Especially, the CoVIC constructs that share the mesa/valley area in binding footprints show competition with communities targeting other RBD binding sites. It is therefore likely not optimal to pair mesa/valley targeting antibodies with other RBD-targeting antibodies for cooperative binding. Interestingly, Adintrevimab (ADG-20), a clinical mAb currently under evaluation, uses the inner-mesa interface (69). By contrast, cryptic site and top targeting antibodies are the two groups that showed the least amount of competition with each other while both generally strongly blocked ACE-2 interaction.

In addition to footprint proximity, RBD is both linked to and surrounded by other domains. Unlike an isolated RBD molecule, cooperative binding on RBD in the context of trimeric spike protein may require more than non-overlapping epitopes. Not only can the closed state of RBD preclude binding access to the cryptic site, but the open state of RBD could restrict the access to the outer face. This may explain the competition between some mesa/valley targeting constructs and outer face targeting constructs. Some antibodies targeting the outer face of RBD were shown to lock RBD in the closed conformation (36, 42). Therefore, some outer face RBD binders can potentially prevent RBD binders targeting other footprints from accessing preferred binding sites, including the cryptic-site.

Antibody constructs that bind to the cryptic site of RBD make up a unique group. The majority of cryptic-site binders effectively block ACE-2 and consistently show enhanced binding and retained neutralization to the B.1.351 variant, with some maintaining a reasonable level of binding to the BA.1 variant (Fig. 4d). Notably, some cryptic-site binders showed neutralization activity without blocking ACE-2 (70). Based on the competition profile, these antibody constructs very likely bind cooperatively with top-RBD-targeting antibodies. Currently, no clinical mAb that received EUA previously from the FDA is known to target the cryptic site (44, 45), likely in part due to the weaker binding affinities compared to other RBD binding footprints, which, in turn, may associate with weak neutralization activity. However, the binding of cryptic-site-targeting antibodies could be enhanced through molecular engineering, for example, into a multivalent antibody construct. Strategies of this type such as creating multimers from VHH were used by others and were shown to retain neutralization to multiple VOCs including Omicron (71).

Interestingly, most of the NTD-specific antibody constructs in this panel lost the ability to bind to B.1.351 HexaPro, with only 11 out of the 43 NTD-specific constructs within CoVIC 1–405 showing enhanced affinity or less than threefold decreased affinity (Fig. S7). It was shown that the majority of the neutralizing activity in convalescent patients is contributed by RBD targeting antibodies (18, 68, 72). However, the B.1.351 variant seems to have also evolved to escape binding and neutralization by NTD-targeting antibodies. This highlights the need to expand research on the importance of virus neutralization by NTD targeting antibodies.

While each type of binding footprint presented an overall trend in affinity for the B.1.351 VOC, it is worth noting that select constructs from each binding footprint type retained binding affinity and neutralization activity against VOCs. For example, while antibody constructs targeting the top of RBD show prevalently weakened binding to the B.1.351 variant, a few constructs are escape profile “outliers” and retained or even had enhanced binding, possibly due to distinct residue contacts on the epitope. Escape profile “outliers” can be observed in other communities in the CoVIC panel as well. It was shown by others that antibodies with similar epitope footprints can have very different escape profiles due to differences in key residue contacts (73, 74). Structural investigation of these resistant antibody constructs in the future can reveal binding interactions that are less affected by VOC mutations.

### Conclusion

In this work, we provide a new way to examine epitope footprints on RBD and NTD based on the HexaPro binning profile and have shown that cooperative antibody binding on RBD requires more than non-overlapping binding surfaces. Among the different binding footprints, antibodies sharing the mesa/valley area as binding sites are not very likely to bind cooperatively with other RBD binders. The antibodies binding to the cryptic site of RBD consistently resist mutations in the B.1.351 variant and show a certain level of affinity retention toward the BA.1 variant which can be particularly explored further. We also showed that antibodies that retain neutralization of the B.1.351 variant are those with retained affinities for B.1.351 HexaPro compared to D614 HexaPro and that Fab binding affinities for D614 HexaPro directly correlate with neutralization of the WT virus. In each type of binding footprint, select constructs retain binding and neutralization toward the B.1.351 VOC. These insights are useful in providing guidance for prevention and treatment options for potential future sarbecovirus outbreaks.

## MATERIALS AND METHODS

### High-throughput SPR binding kinetics

The procedure for measuring CoVIC antibody construct binding kinetics was described previously (54). Briefly, the measurements were done on the Carterra LSA platform using HC30M sensor chips (Carterra) at 25°C, with a single analyte titrated against multiple CoVIC antibody constructs in each assay. Antibody constructs were either captured by amine-coupled goat anti-Human IgG Fc secondary antibody (Millipore) or directly amine-coupled to sensor chips depending on whether the constructs were monoclonal IgG antibodies or not. Antigens were injected in a twofold dilution series onto the chip surface from the lowest to the highest concentration without regeneration, preceded by several injections of buffer for signal stabilization. The highest concentration used for each antigen construct was as follows: RBD 40 µg/mL (1.11 µM), NTD 320 µg/mL (5.71 µM), D614-HexaPro 100 µg/mL (0.181 µM), D614G-HexaPro 100 µg/mL (0.170 µM), B.1.351-HexaPro 100 µg/mL (0.170 µM) ,and BA.1-HexaPro 200 µg/mL (0.351 µM). For each concentration, the cycle times included 120 seconds of baseline, 300 seconds of association, and 900 seconds of dissociation.

The titration data were pre-processed using the Kinetics (Carterra) software before exporting, and then analyzed using the TitrationAnalysis tool developed in-house (75). For each CoVIC antibody construct–antigen pair, the averaged *k_a_, k_d_*, and *K_D_* values from 1:1 Langmuir model fitting were calculated for the best triplicate measurements satisfying the preset data acceptance criteria: (i) standard error of the estimated *k_a_, k_d_*, and *K_D_* in each replicate was ≤20% and 2) the fold change for all three parameters within the triplicate was ≤3.

### ACE-2 blocking assay

ACE-2 blocking assay procedure was described previously (54). Briefly, measurements were done using Biolayer Interferometry (BLI) on an Octet HTX instrument (Sartorius). The data were analyzed using Data Analysis HT 12.0 (CFR11) software (Sartorius). SARS-CoV-2 RBD was covalently immobilized onto Amine Reactive 2nd Generation (AR2G) biosensors (Sartorius), with Human Serum Albumin (HSA) as a parallel reference. Antibody and ACE-2 binding were then performed sequentially. ACE-2 binding to immobilized RBD was monitored in real time in the absence and presence of antibodies pre-bound to RBD. ACE-2 blocking assays for CoVIC 240–269 were performed using a recombinant double-strep tagged ACE-2 construct after performing a bridging assay to ensure consistency between the two ACE-2 constructs. The ACE-2 blocking percentages shown are the mean of triplicate measurements. The percent ACE-2 blocking was calculated as the percentage decrease in ACE-2 binding due to antibodies pre-bound to RBD compared with the ACE-2 binding to RBD in the absence of antibodies. The average response of ACE-2 binding to RBD in the absence of antibody was set as 0% blocking. In each assay, The SARS-CoV-2 Spike Neutralizing mAb (Sino Biological) was used as a positive control. The preset data acceptance criterion was that, if the percent ACE-2 blocking was above the empirically determined lower limit of detection (LLOD) of 13%, the percent CV of triplicate measurements should be under 20%.

### High-throughput SPR epitope binning

Epitope binning was performed with a premix assay format on a Carterra LSA SPR instrument equipped with CMDP sensor chips (Carterra) at 25°C and in a HBSTE running buffer [10 mM 4-(2-hydroxyethyl)-1-piperazineethanesulfonic acid (HEPES) pH 7.4, 150 mM NaCl, 3 mM ethylenediaminetetraacetic acid (EDTA), 0.01% Tween-20]. Two microfluidic modules, a 96-channel printhead (96 PH) and a single flow cell (SFC), were used to deliver samples onto the sensor chip. Surface preparation was performed with 10 mM 2-(N-morpholino)ethanesulfonic acid (MES) pH 5.5 with 0.01% Tween-20 as a running buffer. The chip was activated with a freshly prepared solution of 130 mM 1-ethyl-3-(3-dimethylaminopropyl) carbodiimide (EDC) +33 mM N-hydroxysulfosuccinimide (Sulfo-NHS) in 0.1 M MES pH 5.5 using the SFC. Antibodies were immobilized using the 96 PH for 10 minutes at 10 µg/mL diluted into 10 mM sodium acetate (pH 4.5). Unreactive esters were quenched with a 10-minute injection of 1 M ethanolamine-HCl (pH 8.5) using the SFC. The binning analysis was performed over this array with the HBSTE buffer as the running buffer and sample diluent. The mixture of antibody and D614 HexaPro with a molar ratio of 13.3 (250 nM [37.5 µg/mL] vs 18.8 nM [10.35 µg/mL]) was incubated for at least 30 minutes and then injected in each cycle for 5 minutes, followed immediately by a 2-minute dissociation. The sample injection for every 16th cycle was D614 HexaPro only instead of the antibody-HexaPro mixture. The surface was regenerated each cycle with double pulses (30 seconds per pulse) of 10 mM Glycine pH 2.0.

Data were first processed with Epitope Tool software (Carterra). Briefly, data were referenced using unprinted locations (nearest reference spots) on the array, and the response of each injection cycle was normalized to the response level in D614 HexaPro only cycles. The binding level of the premix mixture just after the end of the injection was compared to and normalized by the binding level of HexaPro alone injections. Signals that show a ratio >0.7 relative to the HexaPro controls are described as sandwiches (76) and represent non-blocking behavior. Competition results were visualized as a heatmap in which red, yellow, and green cells represent blocked, intermediate, and not blocked analyte/ligand pairs, respectively. CoVIC clones that suffered from severe loss of activity or lack of complete dissociation from HexaPro when used as ligands are excluded from the heatmap and further analysis, resulting in a rectangle heatmap. Two separate binning assays were carried out for different subsets of the CoVIC panel, with overlap between the two subsets to ensure continuity. The data for each assay were separated and processed. The two rectangle-normalized heatmaps were then merged into a single heatmap. If a ligand-analyte pair was included in both individual heatmaps, the normalized response from the first binning was used in the merged heatmap.

The clustering analysis of the merged heatmap was carried out in the R environment (Version 4.3.1). First, a square heatmap was created to have all the CoVIC constructs in the merged heatmap appearing both in the ligand direction and the analyte direction: if a ligand-analyte pair has a normalized response in the merged heatmap, then the corresponding value was used in the square heatmap for the ligand-analyte pair; if a pair of antibody constructs had no data as a ligand-analyte pair in the merged heatmap but had data in the reverse orientation (as an analyte-ligand pair), then the normalized data in the reverse orientation were used in the square heatmap as the value for the ligand-analyte direction. The resulting square heatmap was used to calculate the Euclidean distance between each pair of antibody constructs using the “dist” function. Then a dendrogram was generated through hierarchical clustering using the Mcquitty clustering method (77) in the “hclust” function. As a result, clones having similar patterns of competition are clustered together in a dendrogram and can be assigned to shared communities. The original merged rectangle heatmap was then rearranged to visualize the competition patterns of each community.

### Fab production and binding kinetics

A Fab version of CoVIC constructs was either provided directly from the CoVIC headquarters at La Jolla Institute for Immunology following a procedure described previously (54) or produced from IgG in-house. Papain or Lys-C fragmentation was used for cleavage of IgG followed by Protein A Sepharose purification. Reducing and non-reducing protein gel electrophoresis and size exclusion chromatography using Superdex 200 increase column were used to verify the quality and purification of the Fab produced.

SPR titrations were done using the single-cycle kinetics format on a Biacore S200 instrument (Cytiva). During immobilization and titrations, 1× HBS-EP + pH 7.4 was used for the running buffer. Strep-tagged HexaPro constructs were captured onto Streptavidin (SA) chips (Cytiva). Within each kinetics assay, one flow cell channel without immobilized ligand served as a reference channel to monitor and subtract binding responses due to non-specific interactions. Each association step was performed by injecting CoVIC Fab for 120 seconds. Each of the first four association steps was followed by an 80-second dissociation step by injecting a running buffer. The 5th and final association step was followed by at least a 600-s dissociation step by injecting a running buffer. A twofold dilution series of CoVIC Fab was injected from low to high for association steps 1–5. Following the dissociation step, regeneration of the HexaPro surface was performed using of one injection of glycine•HCl pH 1.5 (Cytiva) at 50 µL/min for 30 seconds. The flow rate for association and dissociation was 50 µL/min

The kinetics traces were referenced and subtracted using the reference channel signals and buffer control to obtain antigen-specific binding signals. Then the values for kinetics constants *k_a_*, *k_d_*, and *K_D_* were uniquely determined using Biacore S200 evaluation software.

## Data Availability

All data are included in this article and accompanying supplemental material or can be found at covicdb.lji.org.

## SUPPLEMENTAL MATERIAL

Figures S1 to S7 jvi.01070-23-s0001.pdf Binding kinetics measurements, specificity, epitope binning, communities, affinity correlation, Fab binding kinetics, and KD ratio.

Tables S1 and S2 jvi.01070-23-s0002.xlsx Competition map for HexaPro binning communities of the CoVIC panel, and rate constants and affinity values for select CoVIC constructs as a mAb or Fab binding to D614, B.1.351, and D614G HexaPro.

ASM does not own the copyrights to Supplemental Material that may be linked to, or accessed through, an article. The authors have granted ASM a non-exclusive, world-wide license to publish the Supplemental Material files. Please contact the corresponding author directly for reuse.
