# Supplementary material for: Cryptic-site-specific antibodies to the SARS-CoV-2 receptor binding domain can retain functional binding affinity to spike variants
Source: J Virol. 2023 Nov 29;97(12):e01070-23. doi: 10.1128/jvi.01070-23 (PMC10746274; doi:10.1128/jvi.01070-23)
Supplement: Figures S1 to S7 — Binding kinetics measurements, specificity, epitope binning, communities, affinity correlation, Fab binding kinetics, and KD ratio. [file jvi.01070-23-s0001.pdf]

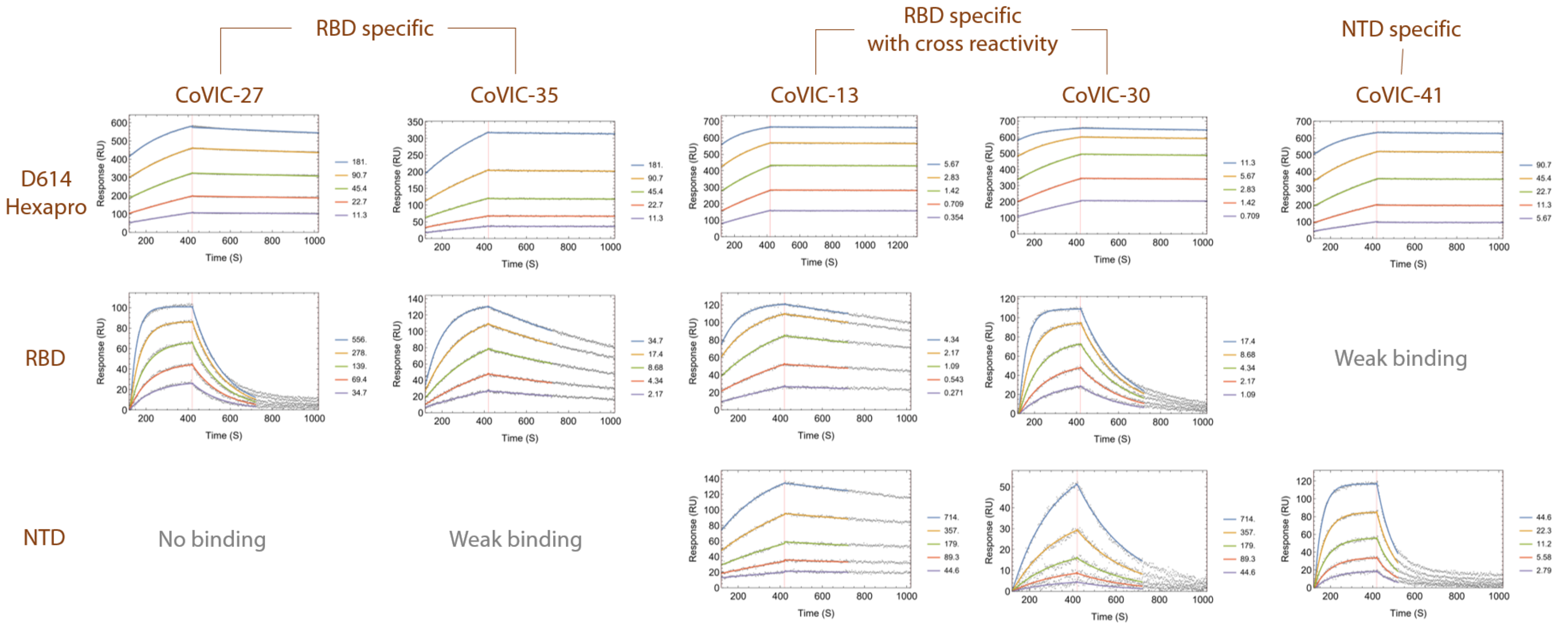

Figure S1: Binding kinetics measurements help determine domain specificity. Sensorgrams of 5 representative CoVIC monoclonal antibodies (mAbs) binding to RBD, NTD and D614 HexaPro. The 5 mAbs represent CoVIC constructs that are RBD specific, RBD specific with cross-reactivity to NTD and NTD specific. In each sensorgram, the color coded fitted curves are overlaid atop the gray underlying data points. The concentration range in nanomolar used for estimating the kinetics parameters is shown in the legend on the right hand side of each sensorgram.

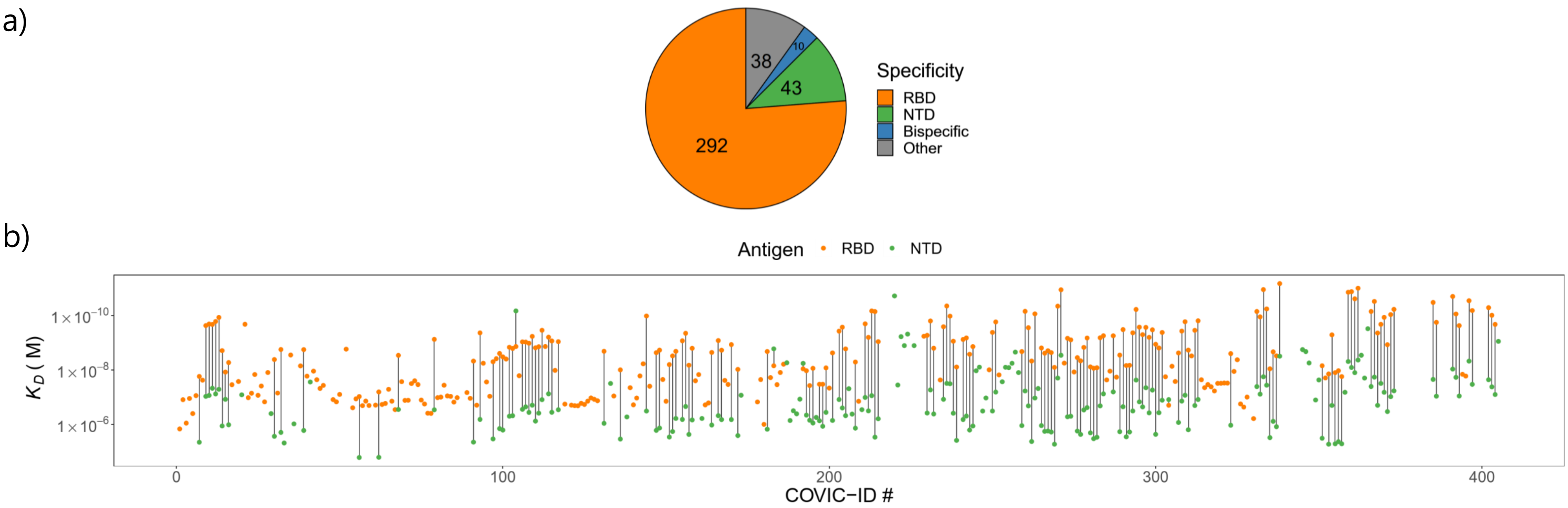

Figure S2: The majority of CoVIC panel antibodies are RBD specific. a) A pie chart showing the number of CoVIC antibodies or antibody constructs for each type of domain specificity. RBD-specific and NTD-specific constructs are represented by orange and green, respectively. For antibody constructs that showed analyzable binding to both RBD and NTD, the domain specificity is determined by comparing the binding affinity to RBD and NTD and defining the construct as specific to the domain for which the binding affinity was more than 10-fold stronger. Alternatively, antibody constructs that are designed to bind both RBD and NTD or that bind RBD and strongly cross react with NTD or vice versa are classified as “Bispecific” (blue). “Other” means the binding site is outside the RBD and NTD. b) The affinity of each CoVIC antibody construct is shown for binding to RBD (orange circle) or NTD (Green circle) or to both RBD and NTD (orange and green circles connected by black line).

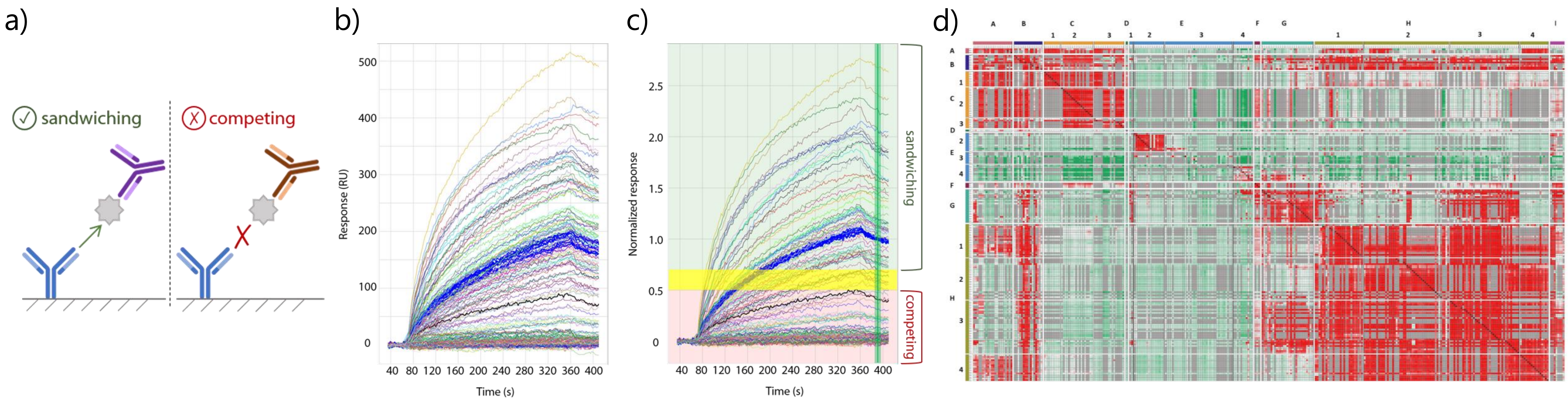

Figure S3: Premix binning assay further defines epitope specificities. a) Cartoon illustration of how sandwiching or competing relationship between two antibodies influences ligand antibody binding to antigen in the premixed complex. b) All the kinetics traces overlaid for an example ligand (CoVIC-3) binding to all applicable premix complexes in the first binning assay. Blue traces represent cycles used for signal normalization in which HexaPro was used as the analyte instead of premix complexes. Black traces represent the competition using premix complex containing CoVIC-3 itself (self-competition). c) Kinetics traces overlaid for CoVIC-3 as the ligand after normalization. The dark green vertical line shows the time point at which the normalized responses were used for defining competition relationships. The green, yellow and red background separate the responses into zones of sandwiching, intermediate and competing, respectively. d) Competition map of HexaPro binning communities from combining the results of two binning assays. Each colored square indicates the normalized response for the specific pair of CoVIC constructs by a gradient color scheme: the most intense red indicates normalized response ≤ 0; the most intense white indicates normalized response = 0.5; the most intense green indicates normalized response ≥ 3; gray indicates data not collected. The CoVIC IDs on the vertical and horizontal direction are respectively the IDs of the ligands (on chip surface) and of the analytes (immune complexed with HexaPro).

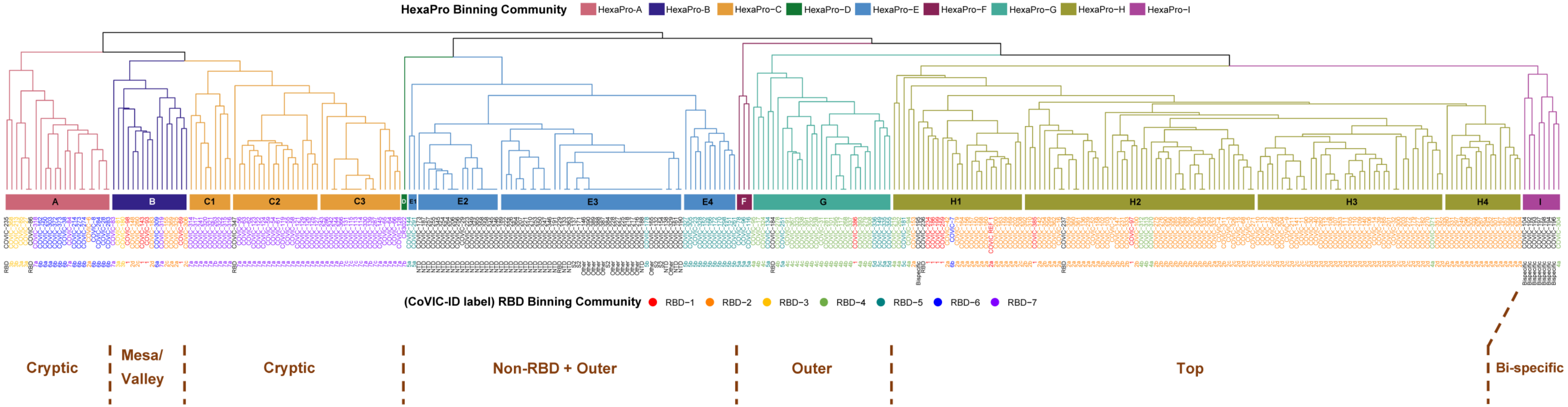

Figure S4: Communities corresponding to constructs targeting the top, outer and cryptic side of RBD match well with RBD communities. Dendrogram of the combined epitope binning assay results using D614 HexaPro as antigen. The naming for each community and the corresponding color in the dendrogram are shown in the legend above the dendrogram (e.g., HexaPro-A, HexaPro-B, etc.). The color of the CoVIC IDs below the dendrogram corresponds to the epitope community assigned from binning with isolated RBD (RBD-1-7) in Hastie et al and Callaway et al (54, 56), with the epitope community or sub-community of each CoVIC ID labeled below the corresponding CoVIC ID. Constructs with black labels either did not bind RBD, or are bispecific, or are RBD-binders that were not included in the RBD binning assay in Hastie et al or Callaway et al (54, 56). Detailed RBD binning community designations are shown under the CoVIC ID labels.

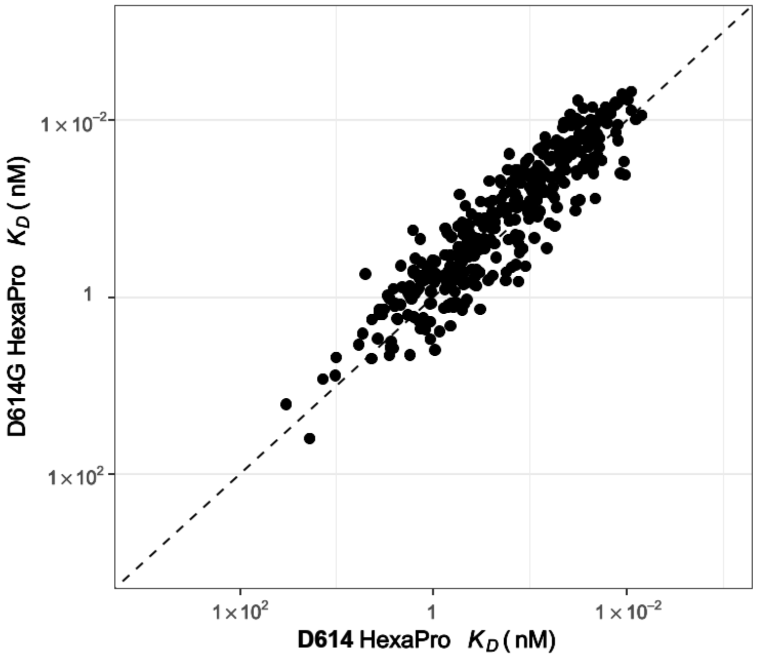

Figure S5: The D614G mutation does not substantially affect the binding affinities of CoVIC constructs for HexaPro. The correlation of K_D values for binding to D614 HexaPro and D614G HexaPro is shown. The diagonal line is a theoretical line at which the correlating affinities are identical. The areas above and below the diagonal line contain CoVIC IDs that have enhanced and weakened affinity, respectively, compared to D614 HexaPro.

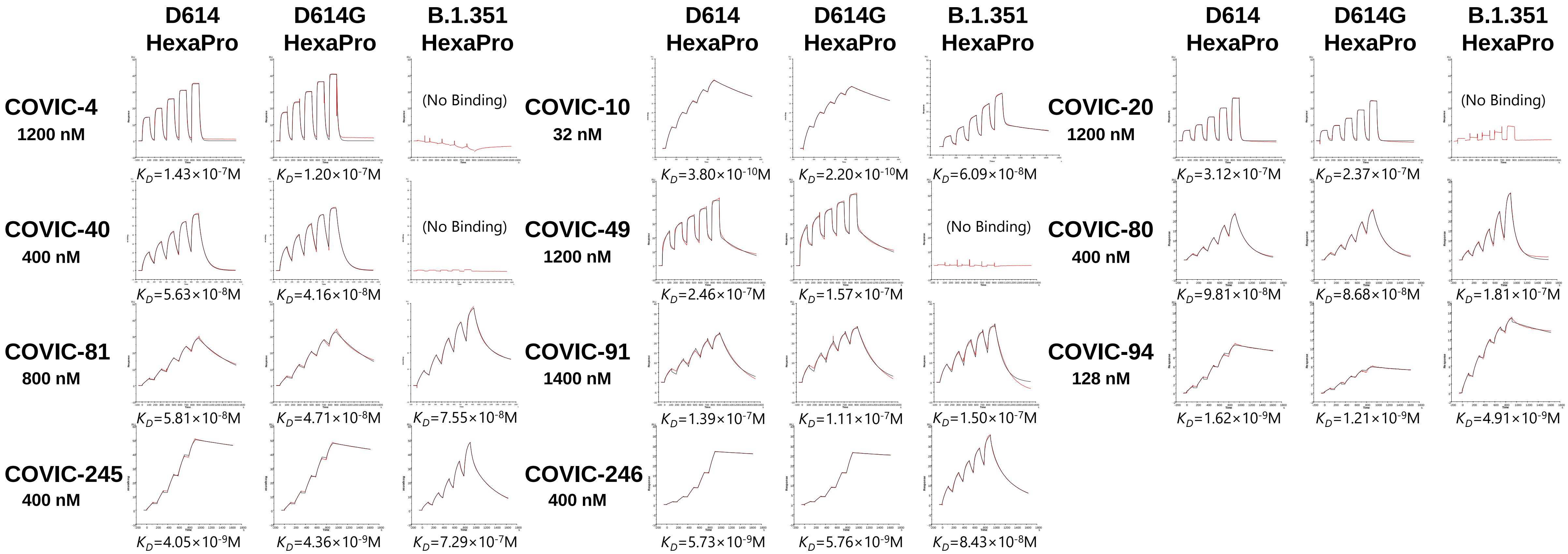

Figure S6: Some CoVIC constructs bound to B.1.351 HexaPro as Fabs. Sensorgrams for 11 CoVIC constructs in Fab form binding to D614 HexaPro, D614G HexaPro and B.1.351 Hexapro are shown. The concentration noted under each CoVIC ID is the highest concentration in the 2-fold dilution series used during the non-regenerative titration for the corresponding CoVIC Fab. The apparent affinity or avidity values were noted below each corresponding sensorgram. “No binding” denotes that Fab form of CoVIC-4, CoVIC-20, CoVIC-40 and CoVIC-49 showed no detectable binding to B.1.351 Hexapro. All sensorgrams shown were collected on a Biacore S200 instrument.

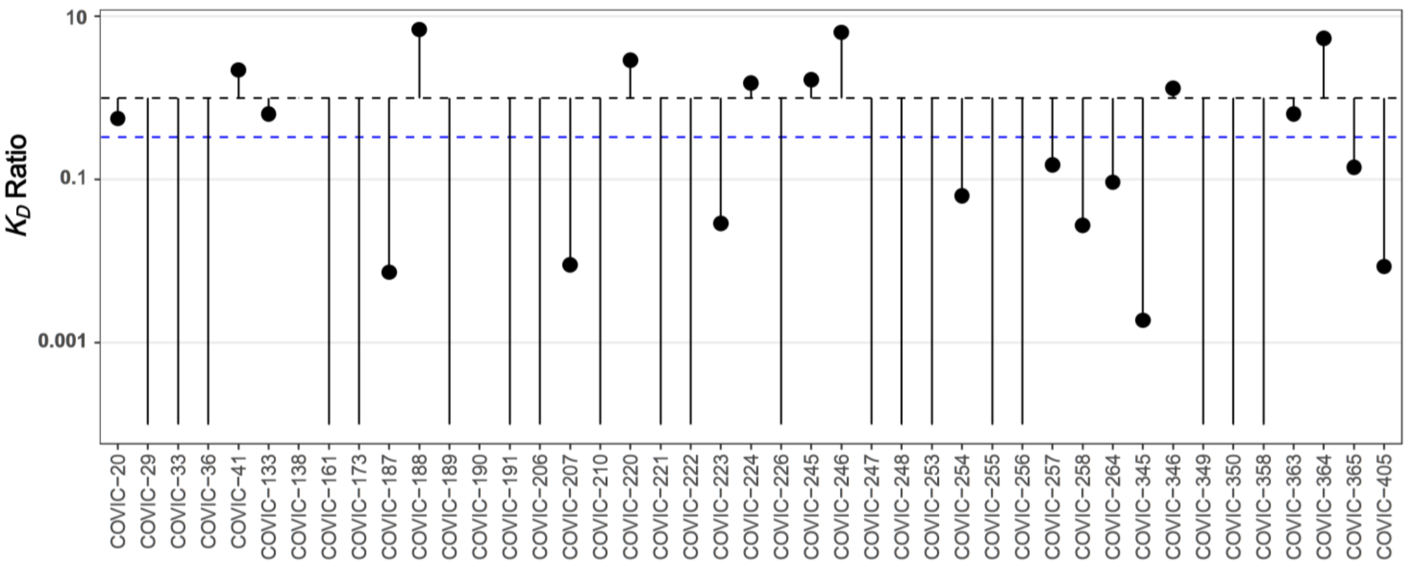

Figure S7: Majority of the NTD-specific CoVIC constructs showed weakened to abolished binding to B.1.351 HexaPro. Affinity ratios (D614 HexaPro K_D / B.1.351 HexaPro K_D) of NTD-specific CoVIC constructs is shown. Each vertical line connects a ratio of 1 to the K_D ratio for a given CoVIC ID. Lines without a capping point correspond to CoVIC constructs that showed no B.1.351 binding. A K_D ratio >1 indicates enhanced affinity for B.1.351 HexaPro relative to D614 HexaPro; K_D ratio <1 indicates lower affinity for B.1.351 HexaPro relative to D614 HexaPro. The blue horizontal line indicates the position at which the affinity ratio is 1/3.
